# Branchial Cilia and Sperm Flagella Recruit Distinct Axonemal Components

**DOI:** 10.1371/journal.pone.0126005

**Published:** 2015-05-11

**Authors:** Alu Konno, Kogiku Shiba, Chunhua Cai, Kazuo Inaba

**Affiliations:** Shimoda Marine Research Center, University of Tsukuba, Shimoda 5-10-1, Shizuoka 415–0025, Japan; University of Hyderabad, INDIA

## Abstract

Eukaryotic cilia and flagella have highly conserved 9 + 2 structures. They are functionally diverged to play cell-type-specific roles even in a multicellular organism. Although their structural components are therefore believed to be common, few studies have investigated the molecular diversity of the protein components of the cilia and flagella in a single organism. Here we carried out a proteomic analysis and compared protein components between branchial cilia and sperm flagella in a marine invertebrate chordate, *Ciona intestinalis*. Distinct feature of protein recruitment in branchial cilia and sperm flagella has been clarified; (1) Isoforms of α- and β-tubulins as well as those of actins are distinctly used in branchial cilia or sperm flagella. (2) Structural components, such as dynein docking complex, tektins and an outer dense fiber protein, are used differently by the cilia and flagella. (3) Sperm flagella are specialized for the cAMP- and Ca^2+^-dependent regulation of outer arm dynein and for energy metabolism by glycolytic enzymes. Our present study clearly demonstrates that flagellar or ciliary proteins are properly recruited according to their function and stability, despite their apparent structural resemblance and conservation.

## Introduction

Cilia and flagella are microtubule-based organelles that are anchored with basal bodies and protrude from the cell surface. They are conventionally distinguished from each other by their lengths, numbers per cell, and/or bending patterns. However, the internal structures and protein components of cilia and flagella are believed to be the same [1, 2]. The internal structure of a motile cilium/flagellum is called an axoneme, which shows a 9 + 2 structure with nine peripheral doublet microtubules and two singlet microtubules at the center. The axoneme is a type of intricate molecular 'machinery' with inner and outer arm dyneins to generate force for beating, and radial spokes and a central apparatus to control the activity of dynein [1, 2].

In vertebrates, motile cilia/flagella play important roles in many biological processes including mucociliary clearance, cerebrospinal fluid flow, tubal transport, sperm transport in efferent ducts, sperm locomotion, and left-right asymmetry of the body [3, 4]. Immotile cilia are specialized for sensory roles and also widely distributed in multicellular organisms. In addition to cilia on sensory cells such as ciliary photoreceptors, olfactory neurons, and hair cells, most non-sensory cells are also able to form immotile primary cilia in vertebrates. Vertebrate primary cilia participate in various critical signaling pathways such as Sonic hedgehog and Wnt signaling [5]. Therefore, dysfunctions of cilia have been shown to cause many diseases, including cystic kidney, Kartagener syndrome, and Burdet-Biedl syndrome [6]. These cilia-related genetic diseases are currently known as "ciliopathies."

Information about the protein composition of cilia is essential to elucidate the versatile nature of cilia and flagella. Comparative genomics [7] and transcriptomics [8] have identified many putative cilia-related genes. More direct proteomic analyses for motile cilia or flagella using cultured human airway epithelial cilia [9], *Chlamydomonas* flagella [10], *Tetrahypena* cilia [11], *Trypanosoma* flagella [12], connecting cilium of mouse photoreceptor cells [13], and rat olfactory cilia [14] have also identified many known and putative ciliary/flagellar proteins and candidate gene products related to human ciliopathies.

Because of the componential and structural conservation of motile axonemes, ciliary research often tends to be focused on the features that are conserved among species. However, it is expected that cilia and flagella found in different tissues in a multicellular organism have differences, since they play a variety of roles depending on tissues or cell types. For example, water-propelling cilia in hamster brain ventricles are not capable of mucociliary transport driven by mucus-propelling cilia in the trachea [15]. Only a few biochemical investigations have reported differences in components among motile axonemes in the same species [16]. Comparative investigations of the components of cilia and flagella in a single species could give us a better understanding of their functional diversity and evolution.

Here we carried out a proteomic comparison of branchial cilia and sperm flagella in the primitive chordate *Ciona intestinalis*. Strikingly, we found that branchial cilia and sperm flagella recruit distinct protein components. These proteins include not only those for regulation of motility, such as those for cAMP- and Ca^2+^-dependent pathway but also basic structural components of axonemes. The present results should shed significant light on the tissue-specific expression and recruitments of axonemal proteins during their formation, as well as the functional diversification of cilia and flagella during evolution, and tissue-specific dysfunction of cilia in ciliopathy [17].

## Materials and Methods

### Chemicals and reagents

IPG strips and buffers were purchased from GE Healthcare (Buckinghamshire, England). Ammonium bicarbonate and Triton X-100 were purchased from Sigma-Aldrich (St. Louis, MO, USA). Trypsin was purchased from Promega (Madison, WI, USA). HCCA and peptide calibration standards were purchased from Bruker Daltonics (Billerica, MA, USA). SDS-PAGE molecular weight standards were purchased from Bio-Rad (Hercules, CA, USA). Anti-acetylated **α**-tubulin antibody (clone 6-11B-1) was purchased from Funakoshi (Tokyo, Japan). Alexa 488-conjugated anti-mouse antibody was purchased from Invitrogen (Carlsbad, CA, USA). OsO_4_, propylene oxide and Quetol 812 were purchased from Nisshin EM Co. (Tokyo, Japan). All the other reagents were purchased from Wako Pure Chemicals (Osaka, Japan) or Nacalai Tesque (Kyoto, Japan).

### Preparation and fractionation of C. intestinalis branchial cilia and sperm flagella

Adult *C*. *intestinalis* were collected from Onagawa Bay near the Onagawa Field Research Center, Tohoku University, or Maizuru Bay near the Maizuru Fishery Research Station, Kyoto University. Aminals were cultured in the sea near these university facilities. These locations are not privately-owned or protected and no specific permits were required for the described field sampling. The branchial wall was carefully sliced without any contamination of mantles and rinsed several times with filtered seawater, then moderately treated in ice-cold hypertonic solution (1.5 M NaCl, 1 mM MgCl_2_, 0.5 mM EGTA, 20 mM Tris-HCl, pH 8.0) and filtrated by mesh (72 μm) to remove tissue and cell debris. Cilia were recovered from the filtrate through successive centrifugation. Sperm were collected directly from the sperm ducts. The sperm suspended in filtered seawater were then homogenized by a Teflon homogenizer to separate the heads and tails. After the homogenate was centrifuged at 500 g for 10 min at 4°C, the supernatant containing flagella was collected. Flagella were purified through further successive centrifugations. Ciliary and flagellar pellets were rinsed twice with 1 M mannitol and processed for 2DE.

### Proteomics

Protein separation, image analysis and quantitation of branchial cilia and sperm flagella were performed as described [18]. Branchial cilia or sperm flagella were suspended in a lysis buffer with an IPG buffer (final concentration 0.5%), and applied to an IPG strip (pH 3–10). Isoelectric focusing was carried out on the Ettan IPGphor III (GE Healthcare) at a programed voltage [18]. Two-dimensional SDS–PAGE was performed using 10% polyacrylamide gels for separating gels. Gels were stained with Coomassie Brilliant Blue R-250 and were scanned using a GS-710 Calibrated Imaging Densitometer (Bio-Rad). Images were analyzed by PDQuest Basic version 8.0 software (Bio-Rad). Spot peaks were automatically detected and manually added or removed. Unique protein spots were assigned with special spot numbers (SSP). The relative quantity (ppm) of a protein spot was expressed as the ratio to the total quantity of all protein spots. At least three gels were analyzed to obtain reproducible 2DE pattern. Comparison and matching of the spot pattern in 2D gels were performed by PDQuest (BioRad). The proteomic identification of protein components in branchial cilia and sperm flagella was carried out by the combination of 2DE and peptide mass finger printing with MALDI-TOF/MS (Bruker Daltonics), as described [18].

### Western blotting

SDS-PAGE was performed as described by Laemmli (1970) [19] with 10% polyacrylamide gels. Proteins transferred onto PVDF membranes were detected by antibodies against several axonemal components. Same amount of proteins from branchial cilia and flagella were loaded, electrophoresed and transferred to PVDF membrane. For comparison of signals between branchial cilia and flagella, blots of their lanes adjacent to each other or those of two lanes from the same PVDF sheet were used for western blotting. Preparations of antibodies have been described for HSP40 [20], IC2, cAMP-dependent protein kinase regulatory subunit II (PKARII) [21], ARMC3 (ARM94) [21], WDR63/IC140 (IC116) [21], Tctex2-related light chain (Tctex2-LC) [21], PF16/SPAG6 [22], calaxin [23], and ODF3/Shippo1 [24]. Anti-tektin 3 antibody was prepared by the method previously described [20]. The following PCR primers were used for amplification of the open reading frame for *Ciona* tektin 3 [5'- GCGCGGATCCATGGAGATAGCCGGTTCA-3' (sense) and 5'- GCGCGAATTCACCGTTCAAAACTTTCCAG-3' (antisense)] PCR was performed using testis cDNA library. The PCR products were subcloned into the pET32a. The expression of tektin 3 was induced by IPTG and purified by Ni immobilized His-Bond metal chelation resin (Novagen, Madison, WI). The mice were given three subcutaneous injections at intervals of 14 days, followed by a booster dose 1 week before serum collection. For secondary antibody, goat anti-mouse IgG (H+L)-HRP (Invitrogen) was used. Signals were detected by the ECL-plus detection system (GE Healthcare).

### Immunofluorescence microscopy and electron microscopy

Branchial walls from adult *C*. *intestinalis* were fixed in a solution containing 4% paraformaldehyde, 0.45 M sucrose, 0.1 M MOPS (pH 7.5) for 2 h at 4°C. The immunofluorescence procedure was the same as that used for *C*. *intestinalis* larvae [21]. For the transmission electron microscopy, branchial wall and sperm were fixed, dehydrated and embedded as described [25].

## Results

### Ultrastructures of branchial cilia and sperm flagella in *Ciona intestinalis*

**Fig 1:**
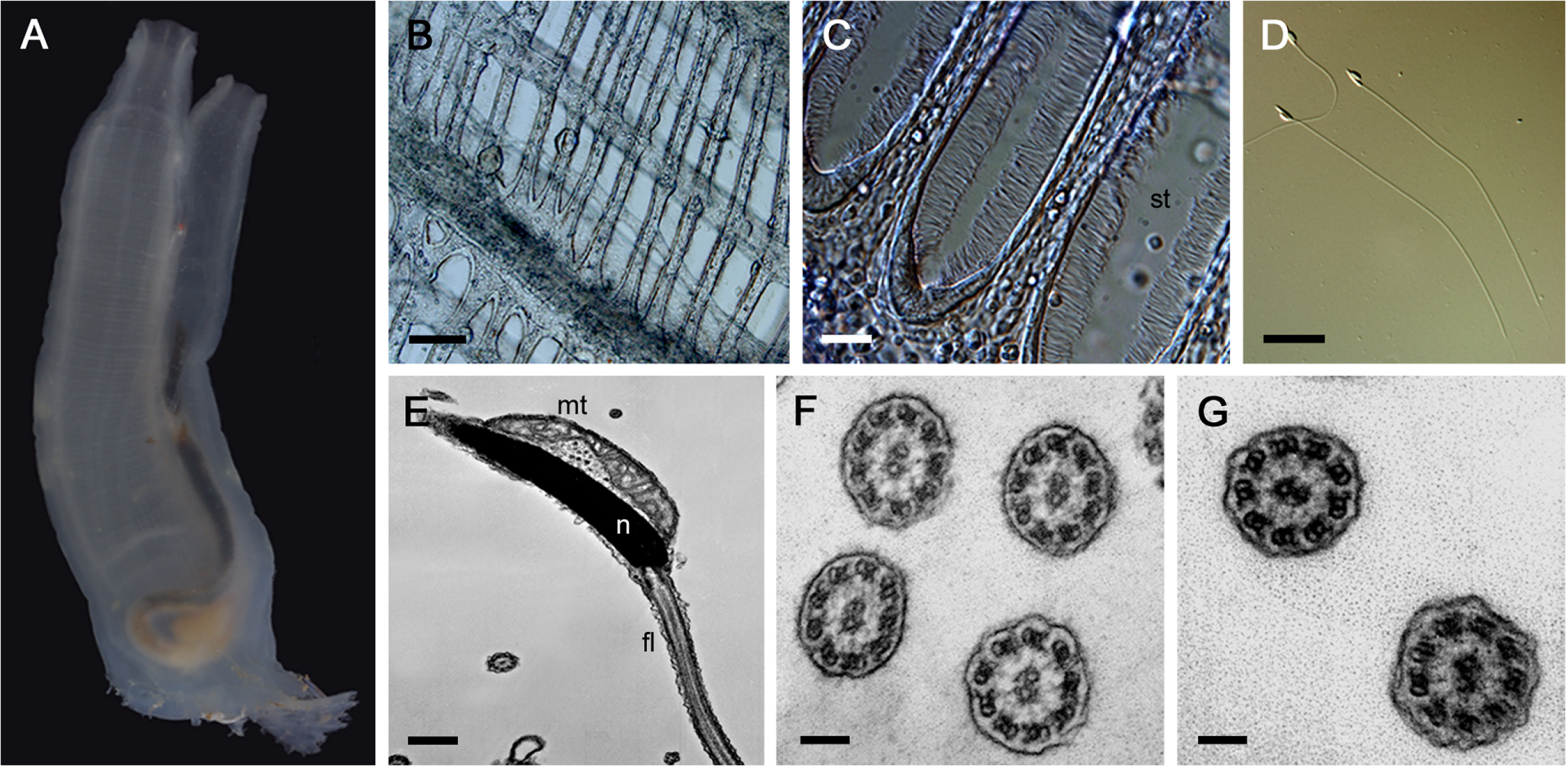
Morphology of branchial cilia and sperm flagella in *Ciona intestinalis*. **A.** An adult *C*. *intestinalis*. **B.** The branchial wall is perforated by eliptical halls called 'gill slits' or 'stigmata.' Scale bar = 100 μm. C. A magnified stigma. st, stigma. Each stigma is lined by ciliated stigmatal cells. Scale bar = 20 μm. **D.** Differential interference contrast (DIC) microscopy image of *Ciona* sperm. Scale bar = 10 μm. **E.** A TEM image of *Ciona* sperm. Only one mitochondrion is attached to the head, and the flagellum does not have any accessory structure. Putative glycogen particles can be seen between the nucleus and mitochondrion. n, nucleus; mt, mitochondrion; f, flagella. Scale bar = 500 nm. **F** and **G**. Transverse sections of branchial cilia (F) and sperm flagella (G). Both axonemes show the typical motile 9 + 2 architecture with outer and inner arem dyneins, radial spokes, and central pair apparatus. Scale bar = 100 nm.

Branchial cilia (5–10 μm in length) of the stigmatal cells propel water from a pharyngeal lumen to an atrium. By this flow, sea water is taken from an inhalant siphon. On the other hand, *C*. *intestinalis* sperm have quite simple structures with flagella that are 60–70 μm in length without any accessory structures seen in mammalian sperm such as a mitochondrial sheath, fibrous sheath, and outer dense fiber (Fig 1) [26]. We first compared the internal ultrastructures of the cilia and flagella by transmission electron microscopy (TEM). Under TEM observation, the branchial cilia and sperm flagella showed no prominent difference in their axonemal 9 + 2 architecture, with both outer and inner arms, radial spokes and a central apparatus, or in their non-axonemal compartments, including plasma membrane and the matrix between the membrane and the axonemes (Fig 1).

### Comparison of protein composition in branchial cilia and sperm flagella

**Fig 2:**
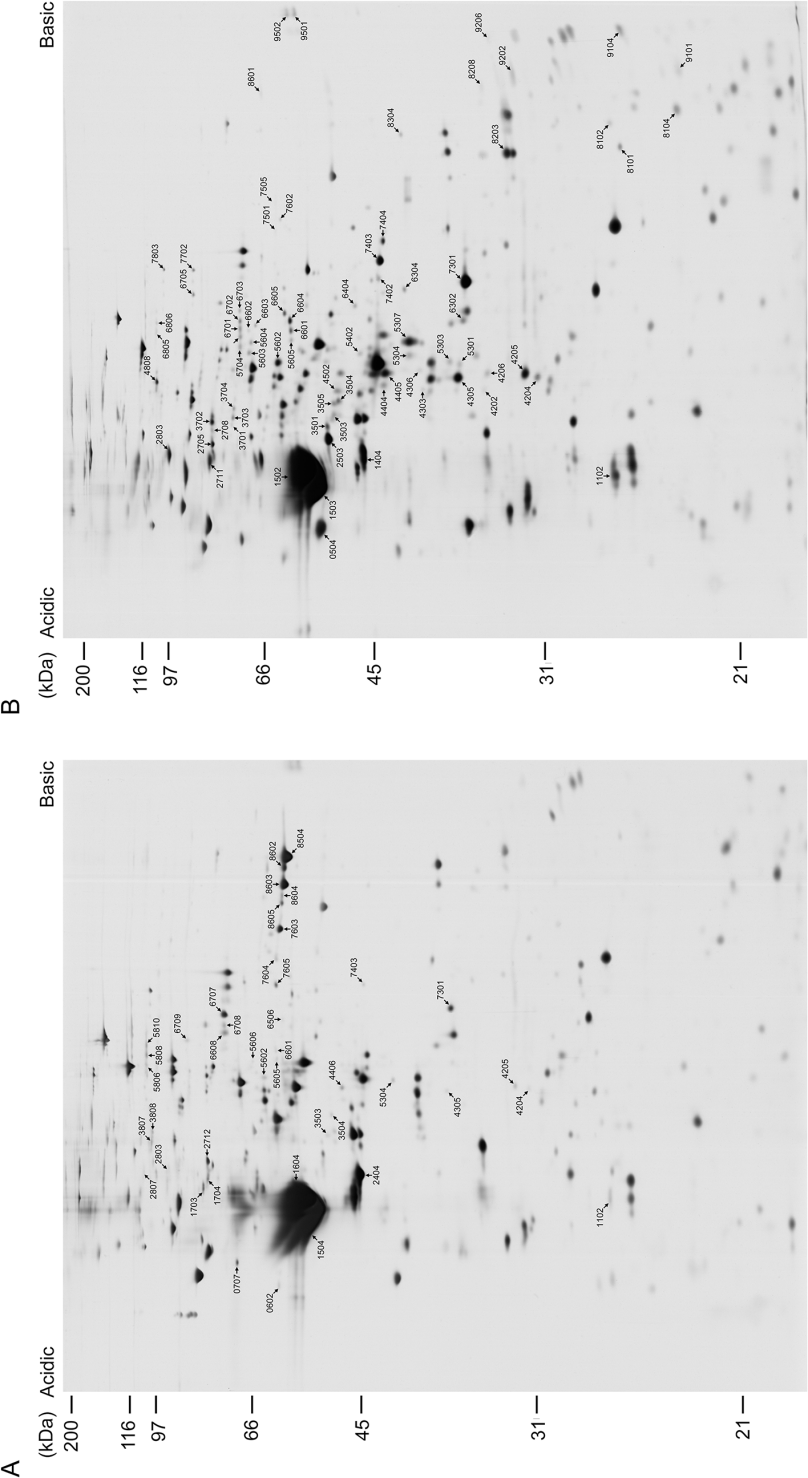
Comparison of 2DE protein patterns between branchial cilia and sperm flagella. Proteins were first separated by isoelectric focusing (pH 3–10), followed by SDS-PAGE with 10% polyacrylamide gel. Proteins were stained with silver. Protein spots predominantly seen in branchial cilia (A) or sperm flagella (B) are indicated by arrows and special spot number, SSP.

To determine whether the branchial cilia and sperm flagella show any differences in components related to cell types, beating patterns, regulation mechanisms, or length, we compared the protein compositions of the branchial cilia and sperm flagella by 2DE with isoelectric focusing (pH 3–10), followed by SDS-polyacrylamide gel electrophoresis (PAGE) (Fig 2 and S1 Fig.). Although their overall electrophoretic patterns were similar in major proteins, the branchial cilia and sperm flagella showed several protein spots at different positions on 2DE gels.

To identify proteins that differ between the branchial cilia and the sperm flagella, we picked up the major protein spots and subjected them to peptide mass fingerprinting (PMF) using matrix-assisted laser desorption-ionization time-of-flight mass spectrometry (MALDI-TOF-MS). To exclude proteins contaminated by other cell components as much as possible, we repeated the analysis three times, and the proteins identified by MS at least twice are specified as ciliary or flagellar components. We identified 486 protein spots in total from branchial cilia and sperm flagella (S1 Table). Protein spots were quantified with the software PDQuest (Bio-Rad), normalized by molecular masses [18] and compared between branchial cilia and sperm flagella. Proteins more than five-times enriched in either branchial cilia or sperm flagella were defined as being present “predominantly” in branchial cilia or sperm flagella (Tables 1 and 2; S3 and S4 Tables). As a result, we identified 83 proteins shared in branchial cilia and sperm flagella (S2 Table), 18 proteins present predominantly in branchial cilia (Table 1 and S3 Table) and 51 proteins present predominantly in sperm flagella (Table 2 and S4 Table).

**Table 1:** Proteins present predominantly in branchial cilia.^a^

Category	Normalized Quantity		f/c ^b^	Protein	*Hs* ^c^	*Cr*	*At*
	flagella	cilia					
Cytoskeleton	0	121190	-	alpha-tubulin (TUBA3)	○	○	○
	0	95323	-	beta-tubulin (TUBB2)	○	○	○
	0	30071	-	actin (Ci-CA7)	○	○	○
Other axonemal structures	0	141373	-	tektin 3	○	○	-
						
Signaling and regulation	0	1827	-	calreticulin	○	○	○
	0	31057	-	heat shock protein 70 cognate	○	○	○
	0	8047	-	protein disulfide isomerase (pdi-2)	○	○	○
	0	2752	-	similar to protein disulfide isomerase	○	-	-
	0	698	-	fascin	○	-	-
Metabolism	0	4568	-	fumarylacetoacetase	○	○	○
	0	1265	-	glutamate dehydrogenase 1	○	○	○
	0	14186	-	transglutaminase	○	-	-
Uncharacterized proteins	0	1798	-	complement component C3	○	-	-
	0	3732	-	elongation factor 2	○	○	○
	1743	20944	0.08	tetratricopeptide repeat protein 29	○	○	-
	0	29892	-	EF-hand domain-containing family member B	○	○	-
	0	15844	-	78 kDa glucose-regulated protein	○	○	○
	0	9910	-	chromosome 6 open reading frame 97	○	-	-

^a^ See S3 Table for detailed information.

^b^ Protein spots were detected by 2DE for both branchial cilia and sperm flagella, but either of them could not been identified by MS analysis.

^c^ Presence of putative homolog in *Hs* (*Homo sapiens*), *Cr* (*Chlamydomonas reinhardtii*) or *At* (*Arabidopsis thaliana*).

**Table 2:** Proteins present predominantly in sperm flagella.^a^

Category	Normalized Quantity		f/c ^c^	Protein	*Hs* ^d^	*Cr*	*At*
	flagella	cilia					
Outer arm dynein	57429	0	-	axonemal p66.0 (Axp66.0)	○	○	-
	9122	0	-	DC2-related dynein intermediate chain 5	○	○	-
	84248	14812	5.69	calaxin	○	○	○
Cytoskeleton	596202	0	-	alpha-tubulin (TUBA3)	○	○	○
	360411	0	-	beta-tubulin (TUBB2)	○	○	○
	40638	0	-	actin (Ci-CA8)	○	○	○
Other axonemal structures	2130	0	-	tektin-like (TD20)	○	-	-
	1667	0	-	ODF3 (Shippo1) (TD11)	○	-	-
	2528	0	-	kinesin-like protein KIF9 isoform 2	○	○	○
	13134	0	-	no hit found (TD01)	○	-	-
	37375	4432	8.43	armadillo repeat containing 3 (ARM94)	○	○	○
Signaling and regulation	2256	0	-	cAMP-dependent protein kinase, Cα	○	○	○
	77022	0	-	cAMP-dependent protein kinase, R II, α A	○	○	○
	9944	0	-	enkurin, TRPC channel interacting protein	○	○	-
	12708	0	-	rab GDP dissociation inhibitor alpha	○	○	○
	2118	0	-	TSSK2 (TD12)	○	○	○
	17795	0	-	apoptosis-inducing factor 2	○	○	○
Metabolism	23866	3111	7.67	phosphoglucose isomerase	○	○	○
	9912	0	-	transketolase	○	○	○
	4910	0	-	aconitate hydratase, mitochondrial	○	○	○
	109021	19368	5.63	glyceraldehyde-3-phosphate dehydrogenase	○	○	○
	4373	0	-	Cyt b-c1 complex subunit 2	○	○	○
	21451	1294	16.58	phosphoglycerate kinase	○	○	○
	2422	0	-	isocitrate dehydrogenase 3 (NAD+) α	○	○	○
	864	0	-	glucose-6-phosphate dehydrogenase	○	○	○
	4094	0	-	isocitrate dehydrogenase 3 (NAD+) β	○	○	○
	3216	0	-	glutamate dehydrogenase (NADP(+))	○	○	○
	6068	0	-	prolyl-tRNA synthetase isoform 1	○	○	○
	52429	1029	50.96	glycerol-3-phosphate dehydrogenase 1b ^b^	○	○	○
	15338	0	-	alpha-enolase	○	○	○
	4085	0	-	6-phosphofructokinase, muscle type	○	○	○
	2364	0	-	adenylate kinase isoenzyme 1	○	○	○
	8064	0	-	glycogen phosphorylase	○	○	○
	134589	1833	73.43	fructose bisphosphate aldolase	○	○	○
	6017	0	-	glutaredoxin-1	○	○	○
	18742	764	24.55	creatine kinase Mt-CK3	○	-	-
	35321	2580	13.69	Phosphoglycerate mutase 1	○	○	○
	13334	863	15.44	Purine nucleoside phosphorylase	○	-	-
	28953	3977	7.28	pyruvate kinase, muscle	○	○	○
Uncharacterized proteins	2256	0	-	similar to predicted protein	○	-	-
	23754	0	-	similar to RIKEN cDNA 1700009P17	○	-	-
	27148	2089	13.00	similar to predicted protein ^b^	○	-	-
	1553	0	-	EH domain-containing protein 1	○	-	○
	47650	0	-	similar to Uncharacterized protein C13orf26	○	-	-
	1538	0	-	similar to predicted protein	○	-	-
	3386	0	-	similar to leucine rich repeat containing 51	○	○	-
	27376	0	-	EF-hand domain-containing family member B	○	-	-
	6169	0	-	leucine rich repeat containing 34	○	○	○
	34458	0	-	armadillo repeat containing 4	○	○	○
	8183	0	-	similar to MORN repeat containing 5	○	○	○
	1022	0	-	growth arrest-specific 8	○	○	-

^a^ See S4 Table for detailed information.

^b^ Protein spots were detected by 2DE for both branchial cilia and sperm flagella, but either of them could not been identified by MS analysis.

^c^ Ratio of normalized quantity in sperm flagella against that in branchial cilia.

^d^ Presence of putative homolog in Hs (*Homo sapiens*), Cr (*Chlamydomonas reinhardtii*) or At (*Arabidopsis thaliana*).

Proteins identified by PMF were classified into four categories: axonemal components, signal transduction, metabolism, and uncharacterized. The axonemal proteins were further categorized into six groups according to their localizations and functions: outer arm dynein, inner arm dynein, radial spoke, central apparatus, cytoskeletal elements, and other axonemal structures. Many of the axonemal proteins, including components of outer and inner arm dyneins, radial spokes and central apparatus, were detected in both the branchial cilia and the sperm flagella (S1 Fig and S2 Table). The proteins present predominantly in branchial cilia or sperm flagella include three groups: 1) proteins that are uniquely found only in branchial cilia or sperm flagella, 2) proteins with different isoforms, and 3) proteins that were found in both branchial cilia and sperm flagella but are extremely enriched in one or the other. These groupings were not always strict for proteins with very low content in the axonemes.

The most conspicuous cilia-specific protein was one of the structural proteins, tektin 3, which is interestingly testis-specific tektin in mammals [27]. Molecular chaperones including heat shock proteins, protein isomerase family members, and a few unexpected proteins including complement component C3 and elongation factor 2 were also identified as cilia-specific proteins (Table 1 and S3 Table).

In contrast, proteins present predominantly in the sperm flagella contained many enzymes involved in energy metabolism by glycolysis, including glycogen phosphorylase, phosphoglucose isomerase, glucose-6-phosphate dehydrogenase, 6-phosphofructokinase, fructose bisphosphate aldolase, glyceraldehyde-3-phosphate dehydrogenase (GAPDH), phosphoglycerate kinase, phosphoglycerate mutase, alpha-enolase, and pyruvate kinase. Neither hexokinase nor phosphoglucose mutase was found (Table 2 and S4 Table).

The sperm flagella specifically contained proteins potentially involved in signal transduction for sperm motility (Table 2 and S4 Table), including cAMP-dependent protein kinase, enkurin, testis-specific serine/threonine protein kinase 2 (TSSK2), Rab GDP dissociation inhibitor alpha, and armadillo repeat-containing protein 3 (ARM94) [21]. Some axonemal proteins, including a homolog of outer arm dynein docking complex protein 2 (p66.0 and IC5) [28] and a neuronal calcium sensor associated with outer arm dynein, calaxin [23]—and several structural proteins, including the product of Parkin co-regulated gene (PACRG) or outer dense fiber 3 (ODF3)—were found to be abundant in sperm flagella or were sperm flagella-specific (Table 2). Interestingly, ODF3 was identified in *C*. *intestinalis* sperm flagella, which lack outer dense fiber. Several other proteins with MORN repeat, armadillo repeat, leucine-rich repeat or other functional modules were also flagella-specific.

**Fig 3:**
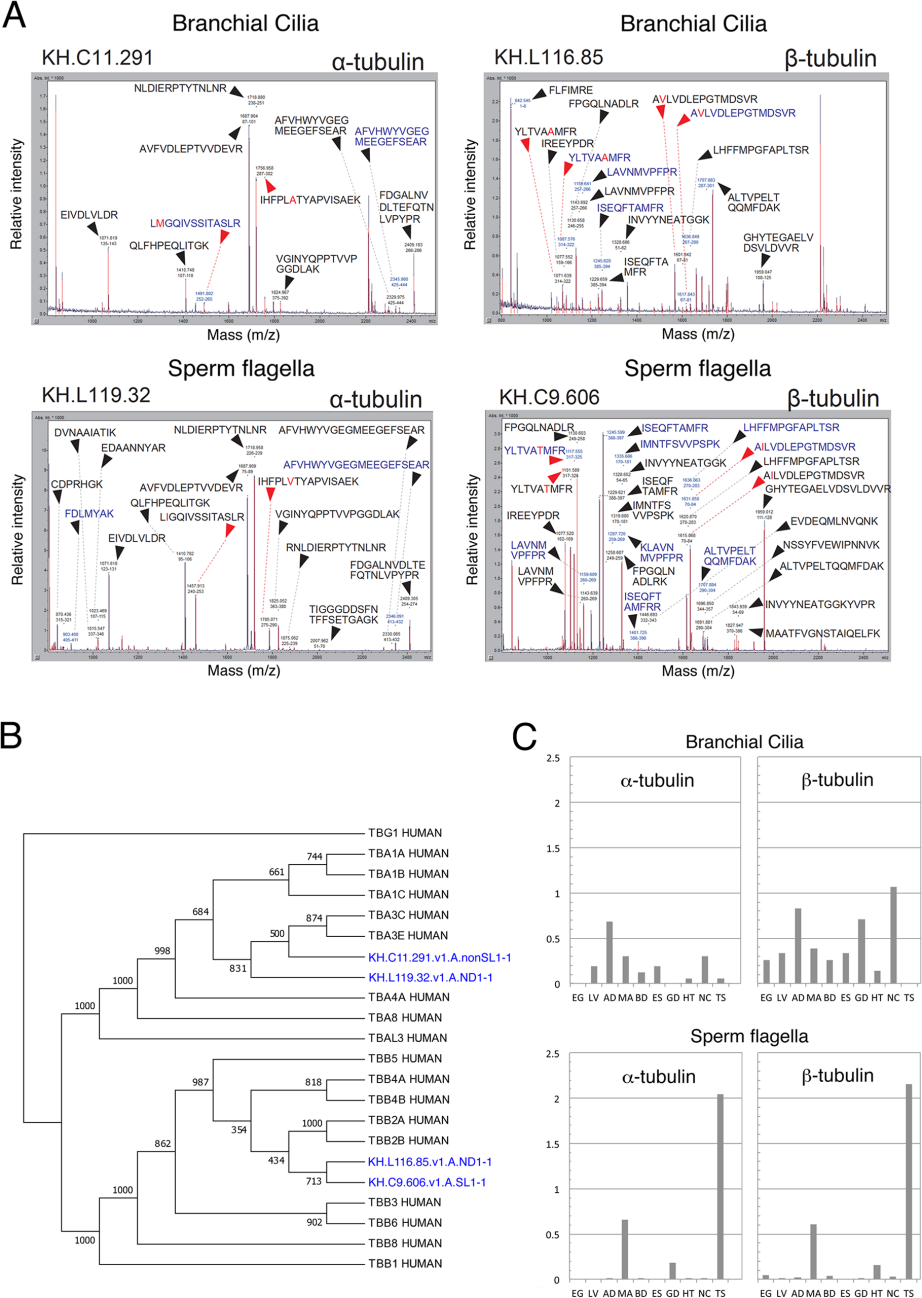
Recruitment of different tubulin isotypes in branchial cilia and sperm flagella. (A) Representative mass spectra of the tubulin spot identified as brancial ciliary α- (KH.C11.291.v1.A.nonSL1-1) and β- (KH.L116.85.v1.A.ND1-1) tubulin and sperm flagellar α- (KH.L119.32.v1.A.ND1-1) and β- (KH.C9.606.v1.A.SL1-1) tubulin. Unique and common mass peaks between the two identifiers are indicated with red and black arrowheads, respectively. The mass value and corresponding peptide sequence are shown on each peak. (B) A phylogenetic tree of tubulin isoforms present in branchial cilia and sperm flagella. *Ciona* proteins (blue) were aligned with human tubulins by CLUSTALW and the tree was constructed by MEGA5. Human γ-tubulin (TBG1) (P23258) was used as the outgroup. The value shown on each branch represents the number of times that a node was supported in 1000 bootstrap pseudo-replications. Accession numbers of protein sequences are: TBA1A (Q71U36), TBA1B (P68363), TBA1C (Q9BQE3), TBA3C (Q13748), TBA3E (Q6PEY2), TBA4A (P68366), TBA8 (Q9NY65), HUMAN, TBAL3 (A6NHL2), TBB5 (P07437), TBB1 (Q9H4B7), TBB2A (Q13885), TBB2B (Q9BVA1), TBB3 (Q13509), TBB4A (P04350), TBB4B (P68371), TBB6 (Q9BUF5), TBB8 (Q3ZCM7). (C) Gene expression pattern of tubulin isoforms in branchial cilia and sperm flagella. Data are based on EST data collected *C*. *intestinalis* tissues (http://hoya.zoology.kyoto-u.ac.jp/). The vertical values in the histogram represent occurrence ratio of the gene against total EST number (percentage). EG, unfertilized egg; LV, larvae; AD, young adult; MA, mature adult; BD, blood cell; ES, endostyle; GD, gonad; HT, heart; MC, neural complex; TS, testis.

It is notable that branchial cilia and sperm flagella were found to use distinct isoforms for major cytoskeletal elements, α- and β-tubulin, and actin. Proteins with gene ID KH.C11.291.v1.A.nonSL1-1, KH.L116.85.v1.A.ND1-1 or KH.L154.4.v1.A.SL1-1 were identified in branchial cilia for α-/β-tubulin or actin, respectively, whereas the corresponding gene ID in the sperm flagella was KH.L119.32.v1.A.ND1-1, KH.C9.606.v1.A.SL1-1 or KH.C3.563.v1.C.SL1-1 (Tables 1 and 2). Although these isoforms have similar amino acid sequences, PMF distinguished them by one or more peptides with a specific sequence (Fig 3A). A phylogenetic analysis of tubulin isoforms in *Ciona* with human tubulins showed that isoforms of α- or β-tubulin in branchial cilia and sperm flagella were grouped into TUBA3 or TUBB2, respectively (Fig 3B). Genes encoding isoforms of α- or β-tubulin in branchial cilia were widely expressed in several *Ciona* tissues, however those in sperm flagella were testis-specific (Fig 3C). Similarly, we found that proteins with similarity to EF-hand domain-containing family member B were contained as different isoforms in the branchial cilia (KH.C9.242.v1.A.ND1-1) and sperm flagella (KH.C8.833.v1.A.ND1-1) (Tables 1 and 2). Taken together, these findings indicate that the branchial cilia and the sperm flagella may use mutually exclusive isoforms for these proteins.

To determine the phylogenetic features of ciliary and flagellar proteins, we carried out a BLASTP search of *C*. *intestinalis* proteins against human, *Chlamydomonas reinhardtii* and *Arabidopsis thaliana*. The homologs showing an E-value less than E-5 are shown in Tables 1 and 2 and in S2, S3 and S4 Tables. Our homology search against the human genome revealed that all of the identified proteins have significantly high homology to putative human orthologs, except for two proteins: hypothetical protein isoform 1 (KH.C11.362.v2.A.SL2-1, no significant homology) and an EF-hand family protein (KH.C7.450.v1.A.ND1-1, E-value = 8E-5). In *C*. *reinhardtii*, 77% (117/152) of the proteins identified showed significant homology, but the structural proteins, including DC2-like intermediate chain (IC4), tektin-like protein, and ODF3 exhibited less homology, as did some metabolic enzymes and uncharacterized proteins.

In contrast, only about 53% (80/152) of the *C*. *intestinalis* proteins identified here were found to have clear homologs in *A*. *thaliana*—mostly metabolic enzymes or those with peculiar domains/modules. Interestingly, some axonemal proteins in *C*. *intestinalis*, particularly those of the radial spokes and the central apparatus, showed significant homology to *A*. *thaliana* proteins, although this organism is non-ciliated.

### Western blotting analysis

**Fig 4:**
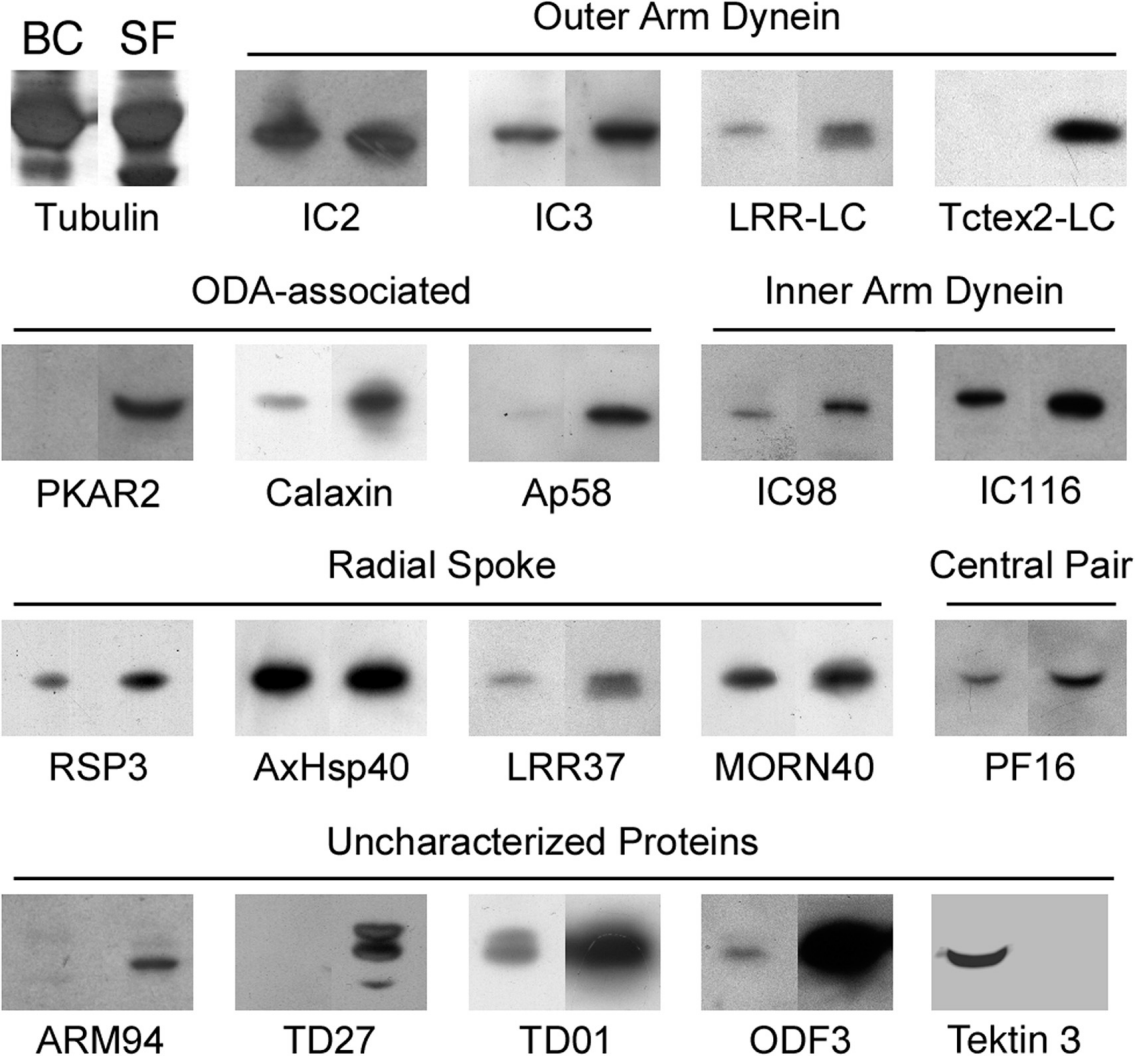
Western blots of cilia and flagella in *C*. *intestinalis* using several antibodies against axonemal proteins. Branchial cilia (BC) and sperm flagella (SF) were separated by SDS-PAGE and immunoblotted with antibodies against several axonemal structures. Both IC2 and IC3 are intermediate chains of the outer arm dynein. LRR-LC and Tctex2-LC are light chains of the outer arm dynein. Ap58 is a protein involved in the anchoring of the outer arm dynein. Both IC98 (ortholog of *Chlamydomonas* IC110) and IC116 (ortholog of *Chlamydomonas* IC140) are intermediate chains of two-headed inner arm f/I1 dynein. RSP3, AxHsp40, LRR37 and MORN40 are components of the radial spoke. PF16 is a protein of the central pair apparatus. PKAR2, Calaxin is a Ca^2+^-binding protein that regulates the outer arm dynein. ARM94 and Tctex2-LC are known to be phosphorylated or dephosphorylated at the activation of sperm motility. ODF3 is a component of the outer dense fiber of mammalian sperm. Both TD27 and TD01 are uncharacterized proteins associated with flagellar axonemes. Tektin 3 is a structural protein in the axoneme (see text).

Our 2DE quantification and mass spectrometry-based identification suggested that several axonemal proteins involved in the regulation of sperm motility were predominantly present in sperm flagella. To further test this, we performed western blotting with antibodies against axonemal proteins in *C*. *intestinalis* (Fig 4). To assess the validity of the quantitative comparison between 2DE gels, we used antibodies that recognize a component of the outer and inner arm dyneins, the radial spokes, and the central pair apparatus that are likely to be contained in almost the same amounts in both the branchial cilia and sperm flagella. An outer arm dynein intermediate chain IC2 [21], a *C*. *reinhardtii* inner arm dynein IC140 ortholog IC116 [21], a radial spoke protein, AxHsp40 [20], and a central pair protein, PF16 [22] were shown to be contained in similar amounts.

The quantitative 2DE analyses of PKAR2, ARM94 and TD01 were further supported by western blots; all three of these proteins were detected exclusively in sperm flagella. Calaxin, which was identified only in sperm flagella by MS, was detected in branchial cilia by western blotting, but only in smaller amounts. We also tested several additional proteins which were not identified by the present MS analysis, with specific antibodies. Tctex2-related dynein light chain (Tctex2-LC) [28] and Ap58 [29] is the regulatory component or associated protein of outer arm dynein, respectively. Western blots showed that both of these proteins are highly sperm flagella-specific. In addition, two uncharacterized axonemal proteins (TD01 and a glutamine-rich 98-kDa protein, termed TD27), the genes of which are both highly expressed in *C*. *intestinalis* testis [24, 30], were revealed to be highly rich in the sperm flagella.

In contrast, quantification on 2DE found that tektin 3 is predominantly present in branchial cilia. Western blotting with anti-tektin 3 antibody detected strong signal in sperm flagella but not in sperm flagella (Fig 4). This clearly confirmed an exclusive expression of this protein in branchial cilia.

### Immunohistochemical comparison

**Fig 5:**
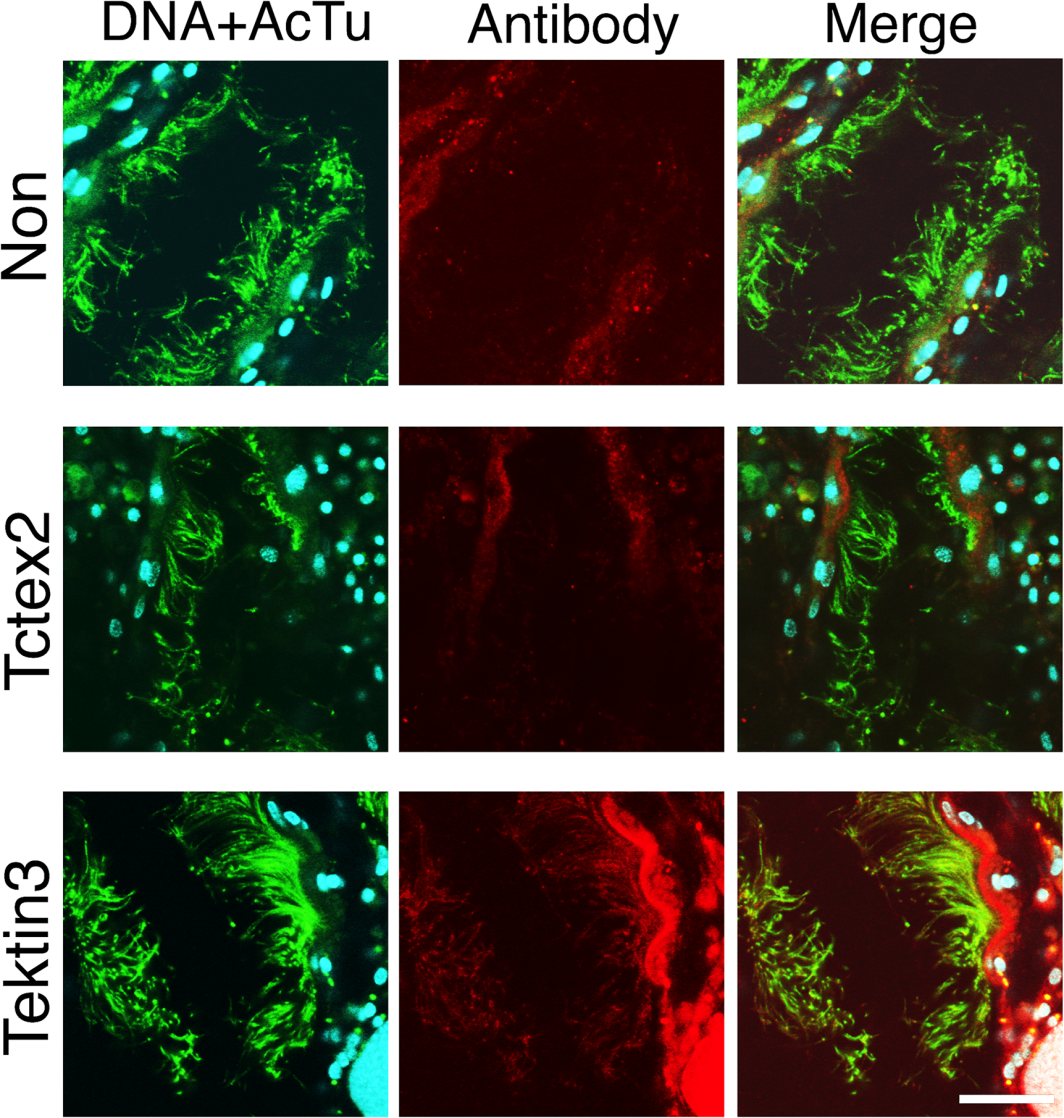
Predominant localization of tektin 3 to branchial cilia. Immunofluorescent microscopy was carried out on trimmed brancial wall with anti-Tctex2 dynein light chain antibody (middle) and anti-tektin 3 antibody (bottom). Non-immune anti-serum was used as a control (top). Samples were stained with anti-acetylated α-tubulin (green), anti-Tctex2 or anti-tektin 3 (red), or 4',6-diamidino-2-phenylindole (DAPI; DNA, blue). Left, double staining with DAPI and anti-acetylated α-tubulin; center, double staining with anti-Tctex2 or anti-tektin 3 antibody; right, merged image from DNA staining, staining with anti-acetylated tubulin antibody and staining with anti-Tctex2 or—tektin 3. Bar, 20 μm.

**Fig 6:**
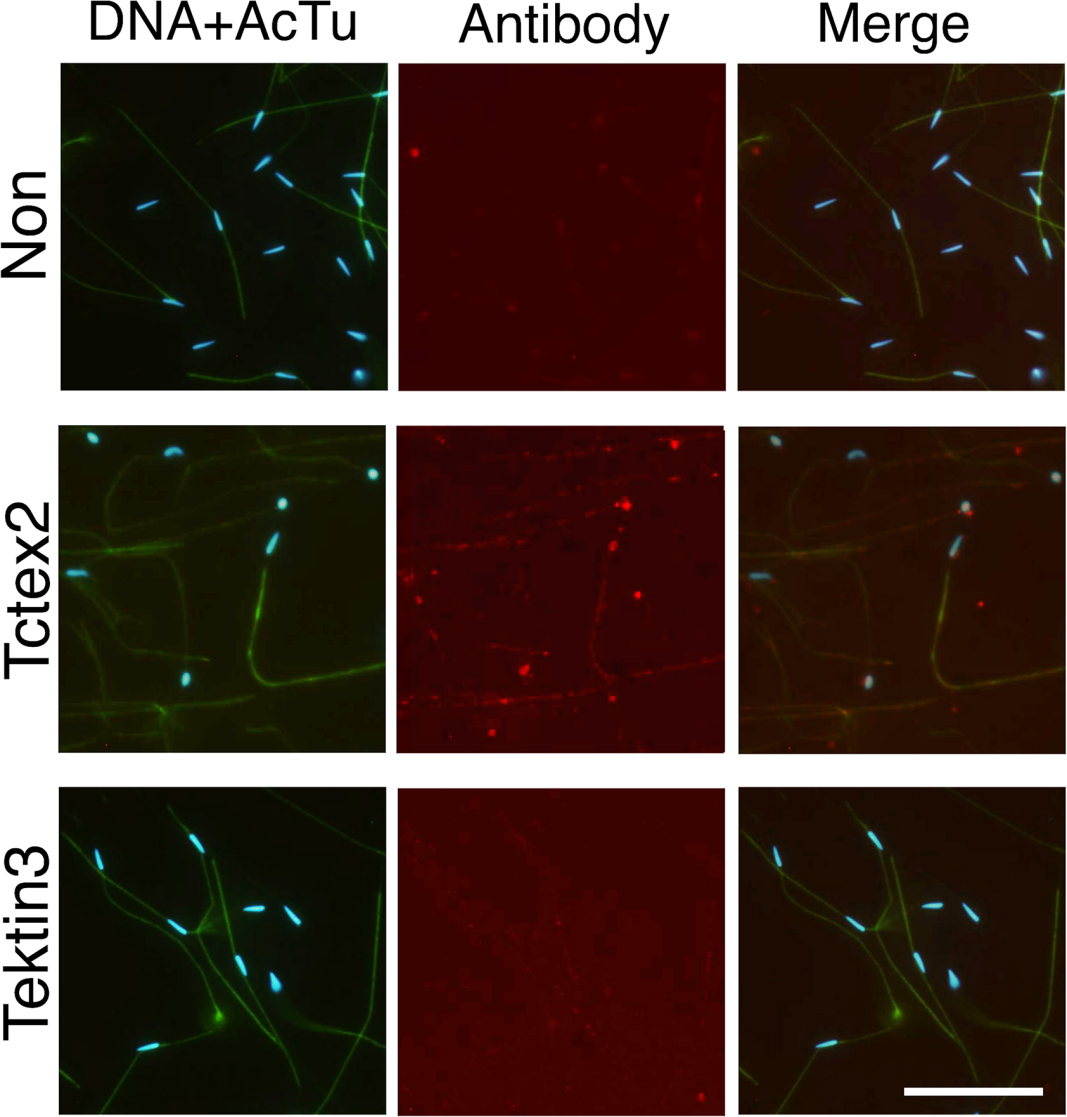
Predominant localization of Tctex2 dynein light chain to sperm flagella. Immunofluorescent microscopy was carried out on sperm attached to the glass slide with anti-Tctex2 dynein light chain antibody (middle) and anti-tektin 3 antibody (bottom). Non-immune anti-serum was used as a control (top). Samples were stained with anti-acetylated α-tubulin (green), anti-Tctex2 or anti-tektin 3 (red), or 4',6-diamidino-2-phenylindole (DAPI; DNA, blue). Left, double staining with DAPI and anti-acetylated α-tubulin; center, double staining with anti-Tctex2 or anti-tektin 3 antibody; right, merged image from DNA staining, staining with anti-acetylated tubulin antibody and staining with anti-Tctex2 or—tektin 3. Bar, 20 μm.

We isolated branchial cilia and sperm flagella by hypertonic salt treatment and homogenization, respectively. It is possible that this difference in isolation methods caused unexpected changes in the protein components of the isolated cilia and flagella. To test the isolation methods for the present analyses, we used immunofluorescent microscopy for Tctex2-LC or tektin 3, which was shown highly predominant in sperm flagella or branchial cilia, respectively, by MS analysis and/or by western blotting (Fig 4). Anti-acetylated α-tubulin antibody showed strong staining of both sperm flagella and the cilia surrounding branchial stigmata. Anti-tektin 3 antibody showed a significant signal along the branchial cilia but not along sperm flagella (Fig 5). In contrast, anti-Tctex2 antibody showed a significant signal along the sperm flagella but not along branchial cilia (Fig 6). These results confirmed the results of our 2DE and western blotting and excluded the possibility for the affect of different isolation methods.

## Discussion

The present study is the first extensive comparison of protein compositions between cilia and flagella from the same species. Previous proteomic analyses of cilia using unicellular organisms such as *C*. *reinhardtii*, *Tetrahymena*, and *Trypanosoma* and multicellular organisms such as mouse and human showed that ciliary proteins are highly conserved from protists to mammals [10–12]. Because motile cilia and flagella are highly conserved in ultrastructural appearance (Fig 1), it has been thought that their structural components are also conserved. In fact, we showed here that many axonemal proteins including those in outer and inner arms, radial spokes, central apparatus, and those with unknown function were shared between branchial cilia and sperm flagella in *C*. *intestinalis* (S2 Table). The present study, however, clearly revealed unexpected differences in protein composition between branchial cilia and sperm flagella. The differences in the components of branchial cilia and sperm flagella involve not only the components for specific properties of sperm flagella such as energy metabolism and the regulation of the dynein motor, but also for the components of the fundamental architecture of the 9 + 2 axonemal structure.

Because of the protein solubility in the lysis buffer for 2D analysis, major part of membrane proteins, particularly transmembrane protein, are considered to be excluded from the present study. Several studies have shown that sensory cilia or sperm flagella contain membrane proteins in relation to their specific function, such as specific odorant receptors and their associated proteins in olfactory sensory cilia [31] and a voltage-dependent Ca^2+^-channel, CatSper, in sperm flagella [32]. For better understanding of the signaling pathway for flagellar or ciliary motility, further proteomic works focused on membrane proteins would be necessary.

### Axonemes of brancial cilia and sperm flagella are constructed from different isoforms of tubulins and other cytoskeletal proteins

It was reported that microtubules of sperm flagella are composed of male germline-specific isoforms of α- and β-tubulin [33]. We found here that branchial cilia and sperm flagella in *C*. *intestinalis* use different isotypes of α- and β-tubulins (Fig 3). Both β-tubulin isotypes in branchial cilia and sperm flagella share a C-terminal axonemal motif (EGEXXX), which is indicated to be important in ciliary/flagellar function [34]. In *Caenorhabditis elegans*, specific tubulin isoforms are not essential for the assembly and function of sensory cilia, although they are important for optimizing functions [35]. Similarly, it is possible that the difference in tubulin isoforms seen in the cilia and flagella of *C*. *intestinalis* is simply a result of haploid-specific gene expression and does not affect the structures or functions. Components of ciliary axonemes undergo turnover through intraflagellar transport [36], whereas sperm flagella are considered stable without turnover. In this sense, tubulin isoforms may be involved in the difference of microtubule stability. It is also possible that distinct isotypes may have different states in post-translational modifications such as phosphorylation, palmitoylation, acetylation, detyrosination/tyrosination, Δ2 modification, polyglycylation, and polyglutamylation, which may also be involved in axonemal stability and protein interaction between tubulin- and microtubule-associated proteins [37].

In the present study, we found that actin isotypes in the *C*. *intestinalis* branchial cilia and sperm flagella were different (Tables 1 and 2). We recently showed that sperm flagella contain testis-specific actin isoform [18]. Major actin in testis, however, is a somatic isotype (KH.154.4), which is the same as that in branchial cilia [18]. In *C*. *reinhardtii*, certain types of inner arm dyneins contain conventional actin as a subunit [38, 39]. We therefore consider both of the actin isoforms seen in *C*. *intestinalis* cilia and flagella to be components of inner arm dyneins. The ratio of the quantities of actin against that of tubulins is much higher in branchial cilia than in sperm flagella (Tables 1 and 2), suggesting that the localization and function of actin are different from those in inner arm dyneins in branchial cilia.

Tektins, first isolated from sea urchin sperm flagella, are axonemal structural proteins conserved among the organisms bearing cilia or flagella [40]. Sea urchin tektins A1, B1 and C1 are related to mammalian tektins 4, 2 and 1, respectively. Mammalian tektin 3 is specifically expressed in testis and is essential to sperm motility [41]. We found tektins 1, 2 and 4 in both branchial cilia and sperm flagella in *C*. *intestinalis* (S2 Table). Unexpectedly, tektin 3 was one of the most abundant structural proteins in the branchial cilia, whereas no spot of it was detected in sperm flagella on the 2DE gels. Tektin 3 has not been identified from either a transcriptional or proteomic profile of airway cilia [9, 42].

We detected KIF9, an ortholog of one of the *C*. *reinhardtii* central pair proteins, KLP1 [43], only in the *C*. *intestinalis* sperm flagella. The 2DE positions where KIF9 spots were found in sperm flagella were occupied by the spots of proteins similar to the human chromosome 6 open reading frame 97 (C6orf97) observed in the branchial cilia (Fig 2). C6orf97 was identified in human airway epithelial cells [42] and appears to be a conserved ciliary protein. It is not clear whether this protein functions in the place of KIF9 in cilia.

### Sperm flagella are specified for cAMP- and Ca^2+^-dependent regulation of motility

We identified several structural and regulatory proteins that are thought to be specialized for fertilization in sperm flagella. The most striking finding is that cAMP-dependent protein kinase (PKA) was highly enriched in sperm flagella (Table 2 and S4 Table). cAMP-dependent protein phosphorylation is well known as an important signal for motility activation in the sperm of several organisms [1, 44]. One of the major targets of PKA is the outer arm dynein, which is involved in the increase of beat frequency, waveform asymmetry and penetration into oocytes [1, 45, 46]. In fact, the regulatory subunit of PKA type II is localized at outer arm dynein [47], participating in the phosphorylation of Tctex2-related dynein light chain at the activation of sperm motility [48]. Calaxin is a Ca^2+^-dependent protein that binds to dynein heavy chain in the presence of Ca^2+^ [23]; the property is essential for sperm chemotaxis [49]. Western blotting show that calaxin is more abundant in *C*. *intestinalis* sperm flagella than branchial cilia (Fig 4), suggesting its function in structures other than the outer arm dyneins. Two proteins for anchoring outer arm dynein to doublet microtubules, IC5 and Axp66.0 [28, 50], were also found to be sperm flagella-specific. In bivalves, it was reported that the subunits of outer arm dyneins that are phosphorylated during motility activation differ between branchial cilia and sperm flagella [51], supporting the idea that the composition and regulation of outer arm dyneins are not the same in branchial cilia and sperm flagella. Moreover, sperm flagella seem more specified than branchial cilia in terms of the regulation of outer arm dynein.

### Proteins in sperm flagella imply ancestral forms of the accessory structures in mammalian sperm

Most parts of mammalian sperm axonemes are surrounded by accessory structures such as the mitochondrial sheath, fibrous sheath and outer dense fiber. Cao et al. [52] identified many proteins in these accessory structures of mouse sperm by proteomic analysis. These accessory structures are suggested to play a "scaffolding" role for signaling proteins and glycolytic enzymes, as well as mechanical reinforcement in flagellar assembly and motility [53, 54]. Although sperm of *C*. *intestinalis* lack these accessory structures, we found several proteins with sequence similarities to known components or associated proteins of the accessory structures of mammalian sperm. Some of these proteins were also found in branchial cilia, including tektins, ropporin 1-like protein, meiosis-specific nuclear structural protein 1, and testis/prostate/placenta-expressed protein isoform 1 (S1 and S2 Tables). We also identified an ortholog of outer dense fiber 3 (ODF3; also known as shippo 1 [55]) in sperm flagella; a very small amount of this ortholog was also present in the branchial cilia (Fig 4).

In addition, some of the glycolytic enzymes including aldolase, GAPDH, phosphoglycerate kinase, and triose-phosphate isomerase are associated with the fibrous sheath in mammalian sperm [54]. A proteomic analysis of *Chlamydomonas* flagella also identified glycolytic enzymes, and at least GAPDH is suggested to be anchored to the axonemes [10]. Although the precise localization of these proteins in *C*. *intestinalis* sperm remains to be determined, our result implies that many components of mammalian sperm accessory structures are derived from axonemes or their associated proteins (Table 2 and S4 Table). It was suggested that sperm accessory structures have been independently innovated in several animal groups, including insects, molluscs, and vertebrates [56, 57]. Although proteins in sperm accessory structures have not been investigated extensively (except for those in vertebrates), studies of the function and localization of these proteins in various animal groups may provide more generalized concepts regarding the evolution of sperm accessory structures.

One of the most conspicuous differences between the branchial cilia and sperm flagella in *C*. *intestinalis* is that the sperm flagella contain quite higher amounts of enzymes involved in energy metabolism. Since most of these enzymes participate in glycolysis and its related pathways, glycolysis seems to play important roles in the energy production for sperm motility in *C*. *intestinalis*. Several studies of mammalian sperm show that glycolysis is more important than oxidative phosphorylation for their motility [53, 54]. In fact, some of these enzymes are tightly associated with the fibrous sheath in mammalian sperm [54]. Since we found in the present study that glycogen phosphorylase is specific to sperm flagella in *C*. *intestinalis*, the substrate for glycolysis appears to be stored as glycogen.

Several studies have described glycogen granules in flagella in many organisms. Our TEM observation of *Ciona* sperm showed electron-dense granules between the mitochondrion and nucleus in the sperm head (Fig 1), and this is thought to be glycogen [58]. Even if this is the case, however, the mechanism of the transport of the substrate to the flagella is unclear. One possibility is that the substrate may be transported with mitochondria during mitochondrial translocation [59].

### Possible explanation for the discordance between cilia and flagella in human ciliopathy

Immotile ciliary syndrome, or primary ciliary dyskinesia (PCD), is a cilia-related genetic disorder in human. Most of them accompany various symptoms, including male fertility, respiratory disorders, situs inversus, and hydrocephalus. One of the characteristics of PCD is a broad range of symptom in disorders in tissues and organs. Because the motile machinery of cilia and flagella, axonemes, are well-conserved structures, it has not been well understood why these discordances among multiple cases occur [17]. From the present study it is clearly shown that the axonemal components are not completely the same between branchial cilia and sperm flagella (Fig 2; Tables 1 and 2). This raises a possibility such that the defect of a protein specific to trachea may causes respiratory dysfunction but not male infertility. In fact, some male patients with deficiency in nasal ciliary beating and abnormality in ultrastructures were reported to show motile sperm with normal ultrastructures [60]. Further studies, in particular analysis of knockout mice showing the discordances in PCD, would shed light on the molecular basis on how the axonemes have become diverse to play specific roles in a variety of cells.

## Supporting Information

S1 Fig 2DE patterns of branchial cilia and sperm flagella with specific spot number (SSP) of shared proteins. Protein spots commonly seen in both branchial cilia (A) and sperm flagella (B) are indicated by arrows and special spot number, SSP.
(TIF)

S1 Table Flagellar and ciliary proteins identified in this study. (XLS)

S2 Table List of proteins commonly present in branchial cilia and sperm flagella. (XLS)

S3 Table Proteins present predominantly in brancial cilia (including other information not appearing in Table 1). (XLS)

S4 Table Proteins present predominantly in sperm flagella (including other information not appearing in Table 2). (XLS)
