# Supplementary figures and images for: Branchial Cilia and Sperm Flagella Recruit Distinct Axonemal Components
Source: PLoS One. 2015 May 11;10(5):e0126005. doi: 10.1371/journal.pone.0126005 (PMC4427456; doi:10.1371/journal.pone.0126005)

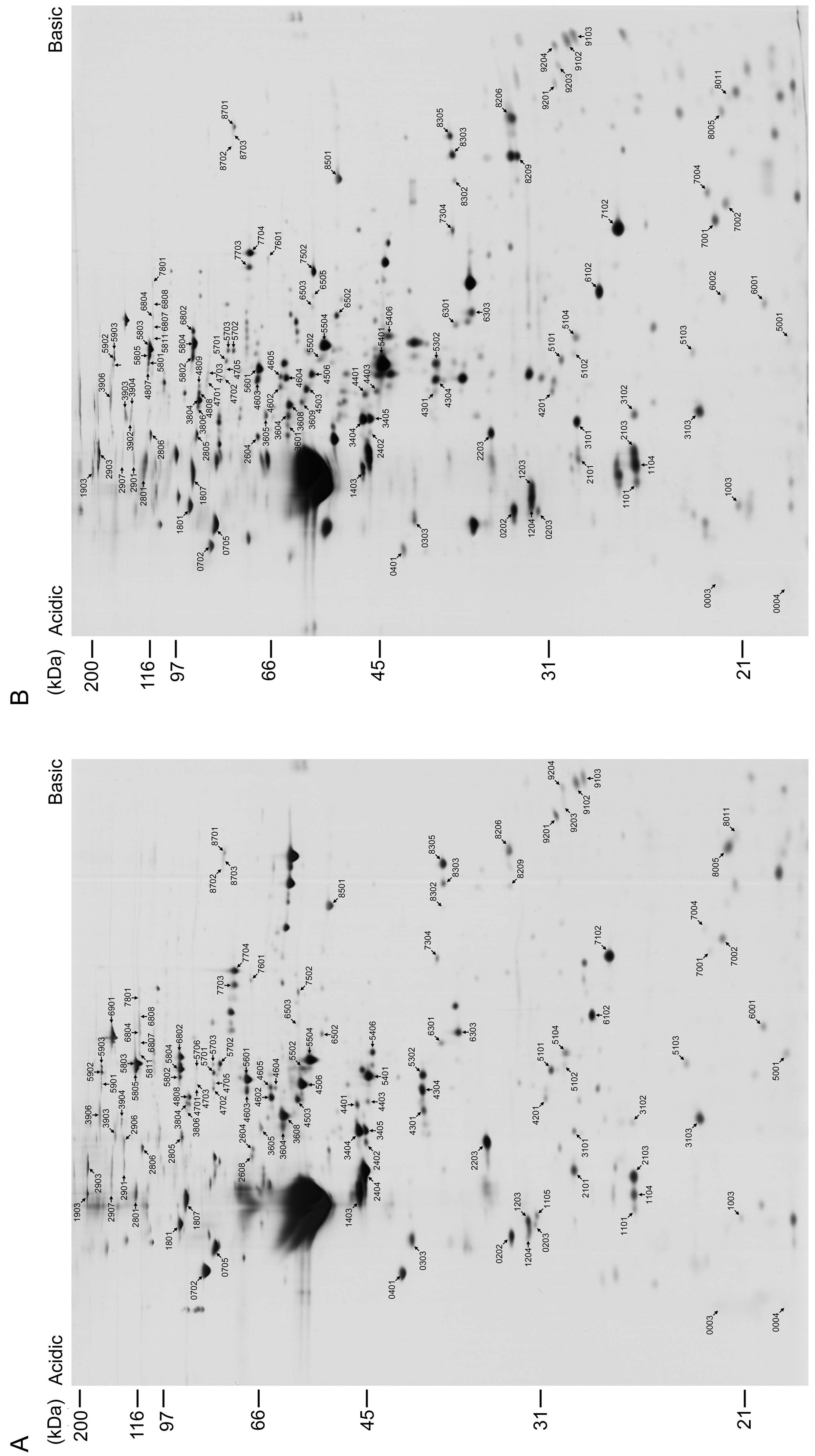

Supplement: S1 Fig — Protein spots commonly seen in both branchial cilia (A) and sperm flagella (B) are indicated by arrows and special spot number, SSP. (TIF) [file pone.0126005.s001.tif]
